# Time to First Analgesic Administration in the Emergency Department for Adolescents/Young Adults With Sickle Cell Disease: A Retrospective Cross-Sectional Study

**DOI:** 10.7759/cureus.77827

**Published:** 2025-01-22

**Authors:** Shreya Kolipaka, Michelle Axe, Brent A Passarello, David Brousseau, Charmaine Wright, Stephanie Guarino

**Affiliations:** 1 Sidney Kimmel Medical College, Thomas Jefferson University, Philadelphia, USA; 2 Community Health Programs, ChristianaCare, Wilmington, USA; 3 Emergency Medicine, ChristianaCare, Wilmington, USA; 4 Pediatrics, Nemours Children's Health, Wilmington, USA; 5 Center for Special Health Care Needs, ChristianaCare, Wilmington, USA; 6 Hematology, ChristianaCare, Wilmington, USA

**Keywords:** acute pain management, acute sickle cell crisis, adolescent/young adult, emergency department utilization, sickle cell disease (scd)

## Abstract

Many adolescent and young adult (AYA) patients with sickle cell disease (SCD) visit the emergency department (ED) for vaso-occlusive crisis (VOC) pain management. Clinical guidelines from the National Heart, Lung, and Blood Institute (NHLBI) suggest that patients should receive an Emergency Severity Index (ESI) of 2 and analgesics within 60 minutes of registration. Despite guidelines, SCD patients experience treatment delays. Our objectives are to describe a Mid-Atlantic healthcare system's guideline adherence and examine the effect of time to first analgesic administration (TFAA) on ED disposition. A retrospective cross-sectional study of the electronic health record data set of ED SCD patient encounters from July 2016 to June 2017 was conducted. Descriptive statistics were run for patient characteristics. ED encounters that followed NHLBI guidelines were included in the data set. Independent t-tests were conducted to examine TFAA differences among patients admitted and not admitted. Majority of encounters were classified as ESI 3 (75.6%), and the mean TFAA was 108.9 minutes. Of ED encounters, 23.9% followed ESI guidelines, 21.5% followed TFAA guidelines, and 4.1% followed both NHLBI guidelines. There was a nearly significant difference in mean TFAA among patients discharged from the ED and admitted to the hospital, a mean difference of -24.5 minutes (t=-1.925; p=0.056). Despite existing ED guidelines, SCD patients experience delayed and variable care. Our results show faster TFAA could potentially decrease admission rates.

## Introduction

Sickle cell disease (SCD) is the most common inherited hemoglobinopathy [1,2]. Due to a congenital abnormality of the oxygen-carrying hemoglobin molecule, under certain conditions, red blood cells will sickle or change shape due to hemoglobin polymerization, making them sticky and fragile [1,2]. Because of the red cell shape, individuals with SCD experience various complications, most commonly painful episodes called vaso-occlusive crisis (VOC) [3]. In a crisis, a considerable number of these individuals, especially adolescent and young adult (AYA) patients, frequently utilize the emergency departments (ED) with pain that is unmanageable with prescribed home medications [4]. 

Previous research on ED utilization patterns of pediatric and adult patients with SCD from 2005 to 2014 found that utilization was highest among young adults [4]. Increased AYA utilization can be attributed to the lack of systemic and institutional support as patients transition from pediatric to adult care [5]. A study examining SCD AYA utilization during transition found increased reliance on the ED and decreased primary care utilization [6]. 

Clinical guidelines from the National Heart, Lung, and Blood Institute (NHLBI) recommend that ED providers assign patients an Emergency Severity Index (ESI) of 2 and administer analgesics to SCD patients within 60 minutes of triage registration for pain relief [7]. Despite the guidelines, SCD patients experience delays in receiving treatment [8-11]. Several pediatric studies of VOC treatment found that the administration of the first dose of analgesic was significantly delayed beyond the national recommendation [9-11]. A shorter time to first dose of analgesic medication was associated with a shorter ED length of stay, but not a shorter hospital length of stay, for those admitted [12]. Additionally, a shorter interval between the first and second doses of medications was associated with decreased hospitalization [13]. Multiple studies have also consistently reported delays, including patient and parent dissatisfaction with VOC treatment due to delay and undertreatment [14,15]. This delay in treatment can be attributed to various barriers in the ED, such as negative provider attitudes about addiction, inadequate provider knowledge, and other psychosocial factors [8,16-18]. A study analyzing ED provider attitudes found that providers with negative attitudes and who care for a higher volume of patients with SCD were related to lower guideline adherence [19]. The study also reported that adult providers tend to have more negative attitudes compared to pediatric providers, further highlighting the relationship between the increased ED utilization and lack of quality care that adheres to guidelines among the AYA population [19].

Previous studies have suggested that personalized pain pathways or standardized protocols in the ED that follow national guidelines significantly improve the analgesic administration time, which leads to improved overall care and patient outcomes [8,20,21]. These studies have also reported decreased admission rates, decreased length of stay, and increased ED discharges [8,21]. However, most studies examining guideline adherence, specifically the time to first analgesic administration (TFAA), have focused on the pediatric SCD patient population. There is limited research on the AYA SCD patient population, and results from pediatric studies cannot be applied to the AYA population due to the difference in care and the stresses of the transition period on the population. Research must be extended to the AYA population because of high utilization. 

The objective of this study is to describe ED adherence to the guidelines and to examine the effect of the TFAA on ED disposition among AYA patients with SCD in the main adult healthcare system in Delaware.

## Materials and methods

Study design

A retrospective cross-sectional study of an electronic health record (EHR) data set was conducted. The data set included all ED SCD patient encounters from July 1, 2016, to June 30, 2017, in a large healthcare system in the mid-Atlantic region. ChristianaCare Hospital ED is a level 1 trauma center and Wilmington Hospital ED is a level 3 trauma center, while Middletown ED is a non-trauma center. This study was considered to be IRB-exempt as per institutional determination. 

Inclusion and exclusion criteria

The study population was defined as patients who had an ED visit encounter with a primary or a secondary discharge diagnosis ICD-10 code of D57.XX, which identifies SCD. Patients with a coded diagnosis of sickle cell trait were excluded. The data set included encounters that took place in the ED alone as well as those that ended with patients admitted to observation or inpatient units from the ED.

Data collection

The retrospective data set was created by querying the EHR system (Cerner) using the above inclusion and exclusion criteria. The final data set included only patients who were between 18 and 39 years old at the time of the ED visit date. TFAA was determined based on triage registration time and time of analgesic administration as per the Medication Administration Reconciliation (MAR) in the EHR. ESI designation was obtained from triage registration documentation. 

Statistical analysis

**Figure 1:**
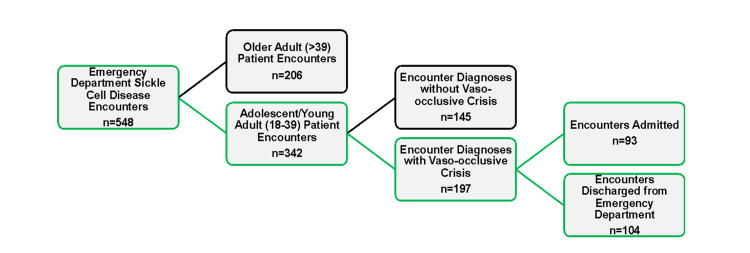
Cohort diagram of ED SCD patient encounters from July 2016 to June 2017 ED: emergency department; SCD: sickle cell disease

The analyses were focused on the AYA ED encounters with VOC, and the final sample size was 197 (Figure 1). Per universal definition, ages 18-39 were considered. Descriptive statistics were run to examine patient characteristics, and the percentages of ED encounters that followed NHLBI guidelines were also determined (ESI 1 or 2 and TFAA ≤60 minutes). Independent t-tests were conducted to examine the differences in TFAA among patients who were and were not admitted.

## Results

Demographics

Of the 197 study encounter patients, 105 (53.3%) were female, with a mean age of 27.3 years (Table 1). One hundred and ninety-six (99.5%) of the patients were Black, and 151 (76.6%) of the patients had Medicaid, Medicare, or dual coverage insurance (Table 1). These rates are consistent with the known demographics of patients with SCD.

**Table 1:** Patient characteristics for ED VOC encounters ED: emergency department; VOC: vaso-occlusive crisis

	n=197
Gender	
Male	92 (46.7%)
Female	105 (53.3%)
Age, mean (years)	27.3
Race	
Black	196 (99.5%)
Other race	0 (0%)
Unavailable	1 (0.5%)
White	0 (0%)
Insurance	
Commercial	57 (28.9%)
Dual	40 (20.3%)
Medicaid	75 (38.1%)
Medicare	19 (9.6%)
Self-pay	3 (3%)

ED encounters and NHLBI guidelines

**Figure 2:**
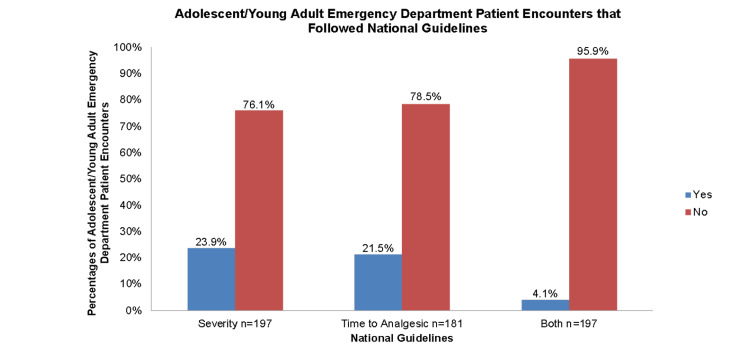
AYA ED patient encounter concordance with national guidelines AYA: adolescent and young adult; ED: emergency department

Among the sample population, the majority of the ED encounters in the health system took place at ChristianaCare Hospital and Wilmington Hospital locations, 98 (49.7%) and 81 (41.1%) of all ED encounters, respectively. Overall, 149 (75.6%) of the SCD encounters were given a severity level of 3. Only 47 (23.9%) of ED encounters followed the ESI recommendations according to the NLHBI guidelines. Additionally, only 42 (21.5%) followed the TFAA guideline recommendation of 60 minutes. Only eight (4.1%) of all encounters in the institution adhered to both national guidelines (Figure 2). Of the total SCD encounters with VOC, 188/197 (95%) had been administered either an opioid analgesic or opioid analgesic combination as the first medication. In 188 encounters, the average time interval between ED registration and analgesic medication administration is 108.9±87.3 minutes (8-460 minutes). There was a nearly significant difference in the mean time to analgesic among patients who were discharged from the ED and those admitted to the hospital, a mean difference of -24.5 minutes (t=-1.925; p=0.056).

## Discussion

VOC is common among AYA SCD patients; thus, utilization of the ED for pain management is frequently seen. Despite existing ED guidelines, SCD patients seen in the EDs of our large community health system experience delays in care and are in pain for long periods of time. Less than 5% of VOC ED encounters followed NHLBI guidelines for pain management. Most of the ED encounters were classified as an ESI of 3, and the mean TFAA was nearly twice the recommendation. Results show that a faster TFAA could potentially decrease admission rates. Patients who were discharged from the ED received an analgesic 24.5 minutes faster than patients who ended up being admitted to the hospital from the ED. These findings support the need for additional pathways and resources to support a faster TFAA as a way to address ED overcrowding and hospital admission rates. These findings, however, might also be influenced by other factors like the severity of VOC which was not explored in this study. 

Other research has demonstrated that patients who receive an ESI designation of 1 or 2 were placed in an ED bed more quickly and had a shorter TFAA. Their average time spent in the ED was shorter compared to patients with SCD and a VOC who received a designation of 3 or higher. Most importantly, this study demonstrated that for every 10-minute increase in TFAA, there was an increase in the relative risk of admission of 0.7% [22]. These findings underscore the need to improve the care of VOC in the ED in order to help health systems achieve the triple aim of decreasing the cost of care, increasing patient satisfaction, and achieving better patient outcomes.

Adherence to national guidelines can potentially improve by implementing a standardized pain pathway or protocol in the ED. The implementation of a pain pathway or protocol in the ED may standardize care, reduce utilization, and improve the overall care of SCD patients. Further research is needed to obtain a deeper understanding of the ED utilization, hospital admissions, and readmission rates among our system's AYA SCD patients. We do know that infusion centers may be an appropriate alternative to treating patients with SCD and VOC. In a multicenter study, research has shown that treatment in an infusion center is associated with a shorter TFAA by almost 70 minutes as well as a decreased rate of admission to the hospital [23]. These findings underscore the opportunity to evaluate other models or sites of care as alternatives to treating patients with SCD and VOC.

Causality cannot be determined due to the cross-sectional design of the study. The number of ED VOC encounters may be under-represented due to diagnosis selection errors in the EHR. There is a potential for medication and time interval inaccuracies as a result of data entry errors and/or inconsistencies. The data may not reflect current times and is only from one Mid-Atlantic community institution. More studies with current relevant data and of more intuitions must be conducted to understand AYA SCD ED treatment and to improve care.

There are some limitations to be noted for this study. Determination of TFAA was based on documentation in the EHR which may not accurately reflect real-time patient experience or accurate timing as sometimes documentation lags due to ED workflow constraints. Additionally, the NLHBI guidelines specify a preference for treatment with intravenous (IV) analgesic medications; however, our analysis used any analgesic medication including those given orally. Although IV medications are ideal, patient preference, ease of obtaining IV access, and staffing may all influence the care delivered to patients. Finally, the identification of encounters to be included in the data set relied on encounter diagnoses which may not adequately capture all relevant patients, inadvertently including or excluding some who would otherwise be appropriate for evaluation. 

## Conclusions

The majority of ED encounters for VOC in this study did not adhere to published NHLBI guidelines for pain management in patients with SCD. The relationship between TFAA and outcomes like hospital admission rates and length of stay should be explored in future work, as our findings suggest that faster administration may be associated with improved patient outcomes. Ways to improve adherence to NHLBI guidelines should be studied in future work through the implementation of standardized pain pathways and protocols in the ED. Further research is needed to create such a standardized pain pathway and to determine the effects on clinical outcomes like length of stay, admission rates, and patient satisfaction.
